# Small-molecule-mediated reprogramming: a silver lining for regenerative medicine

**DOI:** 10.1038/s12276-020-0383-3

**Published:** 2020-02-20

**Authors:** Yohan Kim, Jaemin Jeong, Dongho Choi

**Affiliations:** 1 Department of Surgery, Hanyang University College of Medicine, Seoul, 04763 Korea; 2 HY Indang Center of Regenerative Medicine and Stem Cell Research, Hanyang University, Seoul, 04763 Korea

**Keywords:** Reprogramming, Transdifferentiation

## Abstract

Techniques for reprogramming somatic cells create new opportunities for drug screening, disease modeling, artificial organ development, and cell therapy. The development of reprogramming techniques has grown exponentially since the discovery of induced pluripotent stem cells (iPSCs) by the transduction of four factors (*OCT3/4*, *SOX2*, *c-MYC*, and *KLF4*) in mouse embryonic fibroblasts. Initial studies on iPSCs led to direct-conversion techniques using transcription factors expressed mainly in target cells. However, reprogramming transcription factors with a virus risks integrating viral DNA and can be complicated by oncogenes. To address these problems, many researchers are developing reprogramming methods that use clinically applicable small molecules and growth factors. This review summarizes research trends in reprogramming cells using small molecules and growth factors, including their modes of action.

## Regenerative medicine: Harnessing the reprogramming power of small molecules

The reprogramming of cells using small molecules to generate viable, safe stem-cell populations could transform stem-cell therapies, disease modeling and artificial organ development. Existing ways of reprogramming cells to generate stem cells carry risks, because the methods used often involve using viral DNA components or oncogenes, genes with the potential to turn cells into tumour cells. Safer, inexpensive alternatives are sought by scientists, and the efficient reprogramming of cells using small molecules and growth factors shows promise. Dongho Choi and co-workers at Hanyang University College of Medicine in Seoul, South Korea, reviewed recent research highlighting how small molecules including chemical compounds, plant derivatives and certain approved drugs are being used effectively to create different stem-cell populations. Recent successes are also contributing valuable insights into how stem cells differentiate into different cell types.

## Introduction

Although modern medicine offers treatment methods for many diseases, such as Alzheimer’s disease, heart attacks, and severe liver pathologies, many diseases remain difficult to treat. The most promising mordern technique is stem-cell therapy.

**Fig. 1:**
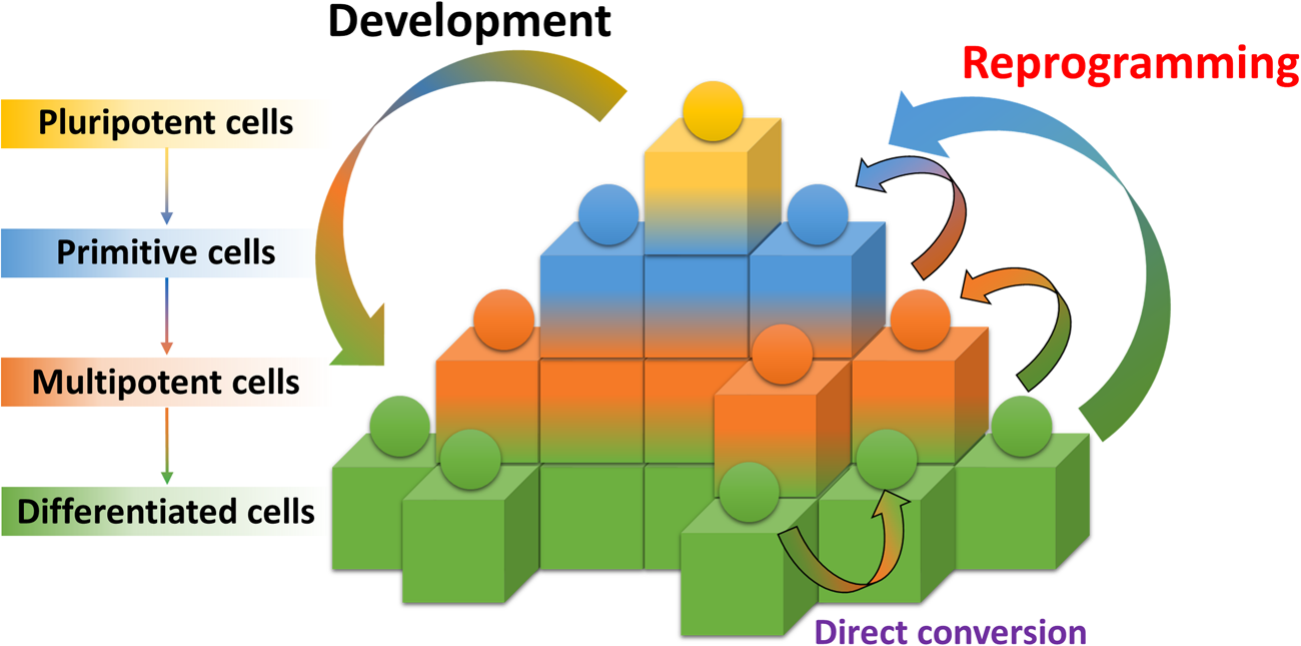
Epigenetic landscape of cellular development and reprogramming. This staircase illustration depicts the fate of cells. The development process begins with pluripotency (yellow) and proceeds through the raw (blue) phase to the multifunctional (orange) and differentiated (green) phases. However, reprogramming can be induced from the differentiated stage to the primitive multipotency stage in response to extrinsic signaling, such as Yamanaka factors. Direct conversion of different cell types at various differentiation stages can be induced by forcing the overexpression of the main transcription factors of the targeted differentiation stage.

In 2006, Yamanaka reprogrammed somatic cells to become induced pluripotent stem cells (iPSCs) using four transcription factors, *OCT4*, *SOX2*, *KLF4*, and *c-MYC*^1^. Human iPSCs were developed using a similar induction strategy^2,3^. Since their induction, iPSCs have been differentiated into neurons^4–7^, pancreatic cells^8,9^, osteogenic cells^9,10^, hematopoietic cells^10,11^, cardiac cells^12,13^, adipocytes^14^, vascular cells^15^, endothelial cells^11^, and hepatocytes^16,17^. Direct conversion, which can transform one cell lineage into another while bypassing the pluripotent stage^18^, became an alternative method for reprogramming cells into neuronal cells^19^ and cardiac cells^20^ in 2010 and into hepatocytes^21^ in 2011 (Fig. 1).

Despite the development of efficient reprogramming, most methods are inappropriate for clinical applications because they carry the risk of integrating exogenous genetic factors or use oncogenes. Alternative approaches, such as those based on miRNA, nonviral genes, nonintegrative vectors, and small molecules, have been studied as possible solutions to the problems^22^.

Among these alternatives, small molecules are attractive options for clinical applications. Reprogramming using small molecules is inexpensive and easy to control in a concentration- and time-dependent manner. It offers a high level of cell permeability, ease of synthesis and standardization, and it is appropriate for mass-producing cells^23^. This review summarizes research efforts involving small-molecule-mediated reprogramming of cells (SmCells) with growth factors from several organs.

## Induced pluripotent stem cells

The efficiency of the first successful efforts to generate iPSCs with four factors was low. Hou et al. conducted studies to improve the efficiency of this process by adding small molecules^24^. Using various small-molecule–screening techniques, they confirmed that the H3K4 demethylation inhibitor tranylcypromine increases the generation efficiency of iPSCs and reported that small-molecule-mediated iPSCs (SmiPSCs) could be generated using only *Oct4*, valproic acid (VPA), CHIR99021, 616452, and tranylcypromine (VC6T). Since then, this group has screened more than 10,000 small molecules to find a replacement for *Oct4* and have found that forskolin (FSK), 2-methyl-5-hydroxytryptamine, and D4476 can replace *Oct4*^24^. They also confirmed that green fluorescent protein (GFP)-positive clusters can be induced using only VC6T and FSK (VC6TF) in an *Oct4*-GFP reporter system. Adding 3-dezanafluronocin A (DZNep), an S-adenosylhomocysteine hydrolase inhibitor, increased the number of GFP-positive cells, demonstrating that FSK can serve as a substitute for *Oct4* and that DZNep enhances the generation efficiency of iPSCs.

Several years later, the same team, Hou et al., addressed the problem posed by a heterogeneous population of fibroblasts that were reprogrammed at relatively low efficiency, and the possibility that a fibroblast subpopulation remains after reprogramming with small molecules^25^. Whether reprogramming through small molecules can be performed in cells derived from ectoderm and endoderm lines was unknown. Therefore, Hou et al. conducted studies of small-molecule–based SmiPSC reprogramming from neural stem cells (NSCs) and small intestinal epithelial cells (IECs), which are ectoderm and endoderm cell types, respectively. First, they confirmed that they could reprogram SmiPSCs using small molecules in nonfibroblasts, and they devised a tracking system with fibroblast-specific protein 1. After confirming the ability to reprogram other cell types, they attempted to reprogram NSCs (ectodermal lineage) with an *Oct4*-GFP reporter system. Attempts to reprogram NSCs into SmiPSCs using the previous VC6TF method had previously failed. However, Hou et al. found that epithelial clusters formed when EPO004777, the inhibitor of the H3K79 histone methyltransferase DOT1L, and Ch55, a retinoid acid receptor (RAR) agonist, were added and the concentration of 616452 was reduced. After adding DZNep to cells cultured for 20 days and switching to a 2i medium consisting of inhibitors of mitogen-activated protein kinase signaling and glycogen synthesis kinase-3 (GSK-3) used in ESC cultures^26^, compact and epithelioid colonies were detected, and the *Oct4*-GFP reporter gradually switched on and caused the formation of SmiPSC colonies. Next, to determine whether small-molecule reprogramming is applicable to endoderm cells, they reprogrammed IECs derived from *Oct4*-GFP transgenic mouse embryos by adding the RAR agonist AM580 to the previously used treatment cocktail followed by VC6TF and DZNep addition after 16 days. Epithelioid clusters were observed between 4 and 8 days after RAR treatment, and colony formation was evident after 15 days. After the conversion to 2i medium on day 40, *Oct4*-GFP-positive ESC-like colonies formed. The team then confirmed that the SmiPSCs generated by combining small molecules had the same characteristics as conventional iPSCs.

For both research projects, the scientific team used GSK-3, TGF-β, histone deacetylase, and monoamine oxidase inhibitors. The successful use of these factors suggests that activation of the Wnt signaling pathway, inhibition of TGF-β signaling, and release of histones are important in the induction of SmiPSCs. Taken together, the data show that small-molecule-mediated reprogramming of all germline lineages into SmiPSCs appears to be possible, suggesting a new way of understanding pluripotency.

## Brain cells

There is currently no effective way to improve a patient’s recovery from nervous system damage. However, recent studies suggest that adult neural stem/progenitor cells can regenerate and function against nervous system damage or disease^27^.

## Neural progenitor cells/neural stem cells

In 2014, Cheng et al. reported that small-molecule-mediated neural progenitor cells (SmNPCs) can be produced from mouse and human fibroblasts using a small-molecule cocktail under hypoxic conditions^28^. They hypothesized that small molecules can induce the endogenous expression of *Sox2*^29,30^ and that the hypoxic state could promote cellular reprogramming^31^. They found that reprogramming somatic cells into SmNPCs from both mice and humans was possible in a hypoxic state of 5% O_2_, with VPA, CHIR99021, and Repsox functioning as the histone deacetylation inhibitor, glycogen synthase kinase, and TGF-β pathway, respectively. The reprogrammed cells expressed NPC-specific markers and were able to differentiate into astrocytes, neurons, and oligodendrocytes.

A few years later, Zhang et al. reported reprogramming mouse fibroblasts into small-molecule-mediated neural stem cells (SmNSCs) using novel small-molecule combinations^32^. They proposed that a combination of small molecules involved in signaling nerve development and targeting epigenetic modifications could induce SmNSCs from somatic cells. They screened small-molecule combinations based on CHIR99021 and fibroblast growth factor (bFGF), which are known to enhance neural development^33,34^, and LDN193189 and A83-01, which are known to inhibit mesoderm and endoderm development^5,35^. In the first screening, they found that Hh-Ag 1.5 (which is a potent small-molecule agonist) and retinoic acid (RA) induced the coexpression of Nestin and Sox2 in the cells at levels of 3.68% and 1.26%, respectively. In follow-up screening studies, they confirmed that RG105 (a DNA methyltransferase inhibitor), parnate (a histone demethylase inhibitor), and SMER28 (an autophagy modulator) increased the number of generated coexpressing cells. Overall, the efficiency of generating coexpressing cells increased by 24.20–30.04% using a combination of 9 small molecules. The SmNSCs produced by this combination were able to proliferate for an extended period, differentiating into astrocytes, neurons, and oligodendrocytes.

## Neuronal cells/neurons

Studies on small-molecule-mediated neuronal cells (SmNs) without a progenitor stage were carried out by Hu et al. in 2015^36^ (comprising the same scientists as the Cheng et al. team^28^), who conducted a screening test based on the results of their previous paper. Initially, human fibroblasts were treated with a combination of VPA, CHIR99021, and Repsox (VCR), which promoted neural differentiation of NPCs. The team did not observe neuron-like cells when they used only VCR. They therefore screened small molecules related to neuronal differentiation and found that a combination of FSK and VCR (VCRF) induced neuron-like cell morphology and the expression of b3-tubulin (TUJ1, a neuronal marker) on day 7. However, the morphology of the cells reprogrammed by VCRF differed from that of typical neurons; round and prominent cell bodies indicated that the reprogramming had been inefficient and partial. To improve the SmN reprogramming strategy, they used additional small molecules and found that a combination of SP600125 (a JNK inhibitor, S), Go 6983 (a PKC inhibitor, G), Y-27632 (a ROCK inhibitor, Y), and VCRF (VCRFSGY) produced TUJ1-positive cells with a neuronal morphology. The cells reprogrammed with VCRFSGY also expressed neuronal markers such as TUJ1, DCX, NEUN, and MAP2 after 7 days. Most of the reprogrammed cells survived for 10–12 days, but neuronal maturation ceased. Subsequently, Hu et al. found that the survival and maturation of reprogrammed cells increased significantly when they added dorsomorphin (an AMPK inhibitor, D) to CHIR99021 and FSK (CFD). More than 80% of the TUJ1-positive cells in these populations expressed vGLUT1, whereas GABAergic, cholinergic, and dopaminergic neurons were rarely found. The electrophysiological properties and gene expression patterns of these cells were similar to those of human iPSC-derived neurons and transcription factor–induced neurons. To determine whether this chemical induction method could be applied to neurological diseases, Hu et al. tested human dermal fibroblasts derived from patients with Alzheimer’s disease. The induced cells derived from the Alzheimer’s patients showed neuronal characteristics similar to those of normal reprogrammed cells. This result offers a new strategy for generating patient-specific neuronal cells and modeling neurological diseases.

At approximately the same time, Zhang et al. reprogrammed human astrocytes into functional SmNs using a small-molecule combination and sequential administration^37^. They had previously reported that glial cells can directly differentiate into neurons through ectopic expression of NeuroD1^38^. They conducted screening studies with 20 candidates that inhibit glial signaling pathways or activate neuronal signaling pathways to find small-molecule combinations that could replace these transcription factors. To increase reprogramming efficiency, they included several small molecules that modify DNA or histone structure. The results from the screening studies conducted with more than 100 conditions at different times and concentrations revealed that a cocktail of nine small molecules could reprogram human astrocytes into SmNs. First, human astrocytes were treated with LDN193189 (0.25 μM), SB431542 (5 μM, a selective ALK5 inhibitor), TTNPB (0.5 μM, a RAR agonist), and thiazovivin (0.5 μM, a ROCK inhibitor, Tzv) for 2 days. Zhang et al. used LDN193189, SB431542, and TTNPB to inhibit glial signaling pathways and activate neural signaling pathways^39–41^ and Tzv to improve reprogramming efficiency and cell survival^42,43^. This initial small-molecule combination was replaced with CHIR99021 (1.5 μM), DAPT (5 μM, γ-secretase inhibitor), and VPA (0.5 mM) 2 days after the initial phase. CHIR99021 and DAPT were used for SmN induction for 4 days^44–46^, and VPA was added to improve the reprogramming efficiency and remained in culture for only 2 days without inducing cell death^29^. For the following 2 days, the team treated the cells with the smoothened agonist (0.1 μM) and purmorphamine (0.1 μM) to activate the sonic Hedgehog signaling pathway, which is the main factor in neural patterning^47^. On day 9, they added the neurotrophic factor neurotrophin 3 and insulin-like growth factor 1 to enhance neuronal maturation. The resulting reprogrammed cells expressed the neuronal markers doublecortin, TUJ1, MAP2, and NEUN and survived for 4–5 months. This reprogramming scheme was not successful for use in spinal astrocytes; it had positive results only in cells originating in the brain. The reprogrammed cells had activity potentials and synchronous burst activity. The researchers then carried out lateral ventricle injections in mice to determine whether the cells could survive in vivo, and they reported that the reprogrammed cells survived in clusters in the brain 1 month after injection. However, the cells failed to undergo reprogramming directly in vivo. These studies show that small-molecule cocktails promote cell plasticity and offer potential for use in transplantation.

Small molecules used in all the papers describing the reprogramming of SmPSCs, SmNSCs, and SmNs activate the Wnt signaling pathway and TGF-β and BMP signaling inhibitors. Wnt signaling is known to stimulate neurogenesis^48^, and inhibition of TGF-β and BMP signaling reportedly induces differentiation of pluripotent cells^30^.

## Cardiac cells

Recently, the generation of cardiomyocytes by direct-conversion techniques, such as the introduction of cardiac transcription factors (including *Gata4*, *Mef2c*, *Tbx5*, and *Hand2*^21,49,50^) or a microRNA combination^51^, has been reported. The teams reported that the overexpression of Yamanaka factor causes heart development in the presence of a JAK inhibitor^52^. A few years later, the same team found that direct conversion into cardiomyocyte-like cells was possible using a combination of *Oct* and small molecules^53^.

Based on those findings and those reported by Hou^24^, Fu et al.^54^ produced the first SmiPSCs using CHIR99021, Repsox, FSK, VPA, Parnate, and TTNPB (termed CRFVPT). Cell clusters similar to cardiomyocytes were developed during SmiPSC reprogramming and beating cells were unintentionally found between 6 and 8 days after treatment with CRFVPT. However, the beating cells were not observed after ~1 week in the SmiPSC-induction condition. Fu et al. therefore used a two-step strategy to promote the stable and effective induction of small-molecule-mediated cardiomyocytes (SmCs), producing a cardiac-reprogramming medium based on the use of CRFVPT at the primary stage. First, they found that bFGF is not required for the generation of the SmCs. They also found that 15% fetal bovine serum (FBS) and 5% knockout serum replacement (KSR) more efficiently generated beating cells than the combination of 10% FBS and 10% KSR that had been used to generate SmiPSCs. Moreover, they added N2 and B27 to increase the induction efficiency. Based on reports that matrix microstructures play important roles in cardiac reprogramming^55^, the scientists conducted the reprogramming in Matrigel-coated dishes, which allowed them to observe more beating cells. Because maintaining a cardiac-reprogramming medium for more than 16 days did not improve the efficiency, they removed the CRFVPT and added CHIR99021, PD0325901 (MEK1/2 inhibitor), LIF, and insulin, which are known maintenance factors for cardiomyocytes^56–58^. As a consequence, they found a significant increase in the number of beating cells. Then, Fu et al. identified the most important factors by removing one compound at a time from the CRFVPT combination, reporting that C, R, F, and V play important roles in the induction of beating cells. They attempted SmC reprogramming of neonatal mouse tail-tip fibroblasts but found that the reprogramming efficiency was lower than it had been for the MEFs. Therefore, they added rolipram (a selective phosphodiesterase-4 inhibitor) to the culture in the primary stage, which increased the reprogramming efficiency. These SmCs expressed cardiomyocyte markers such as α-actinin, cardiac troponin-T (cTnT), cardiac troponin-I, and α-Major Histocompatibility Complex (α-MHC) and accurately exhibited cardiac electrophysiological characteristics. Next, the team confirmed that these cells expressed cardiac precursor markers at an early programming stage and could differentiate into smooth muscle cells and endothelial cells. The results suggest that this reprogramming method was successful because of a cardiac precursor stage similar to one observed during the natural development of myocardial cells.

In the same year, Cao et al. reported reprogramming human fibroblasts into SmCs using nine small-molecule combinations^59^. To facilitate the tracking of SmC reprogramming, they labeled alpha myosin heavy chain-GFP reporters in human foreskin fibroblasts. Guided by the cell activation and signaling-directed conversion paradigm^52,53^, the Cao team used small molecules to induce or enhance cell reprogramming into cardiac cells. First, the researchers conducted screening studies on 89 small molecules known to promote reprogramming. They tested all the combinations against a small-molecule baseline cocktail of SB431542, CHIR99021, Parnate, and FSK, which are known to play important roles in direct conversion of cardiac cells^53^. The cells were treated with various small-molecule combinations for 6 days, after which the treatment was changed to an optimized cardiac-induction medium of activin A, bone morphogenetic protein 4, vascular endothelial growth factor, and CHIR99021 for 5 days. Through these screening studies, they found a cocktail of 15 compounds (15 C) that produced GFP-positive beating clusters. By removing the components of 15 C one by one, they found that CHIR99021, A83-01, BIX01294 (histone methyltransferase inhibitor), AS8351 (epigenetic modulator), SC1 (extracellular signal-regulated kinase 1 and Ras GTPase inhibitor), Y27362 (ROCK inhibitor), and OAC2 (Oct4 activator) were the most important factors. They established that the most efficient combination for reprogramming skin cells into SmCs consisted of seven small molecules and two inhibitors, SU16 and JNJ-10198409. After 30 days of treatment with this small-molecule combination (9 C), the cardiomyocyte marker cTnT was observed in ~6.6% of the cells. The 9 C cocktail also reprogrammed human fetal lung fibroblasts into SmCs. Through a series of processes, the mesoderm, cardiac progenitor cells, and cardiomyocytes are generated during cardiogenesis^60^. This development was also observed in cells treated with 9 C. After treatment with cardiomyocyte induction medium, ~27.9% of the cells expressed KDR, which is an important mesoderm marker. These cells expressed mesoderm, cardiac progenitor cells, and cardiomyocyte genes sequentially over time. Directly converting human cardiomyocyte-like cells using viruses has an extremely low reprogramming efficiency of ~0.1%, and the resulting cells are heterogeneous^61^. However, the production of SmCs produced via 9 C was ~97% efficient, and the homogeneity was confirmed by single-cell quantitative reverse-transcription polymerase chain reaction (qRT-PCR) analysis. Approximately 45–50 days after reprogramming, intercellular electrical recordings showed strong action potential. In addition, most SmCs responded to caffeine, isoproterenol, and carbachol with a similar ventricular activity potential. These reprogrammed cells are therefore functional and have electrophysiological characteristics. The results show that more functional cells can be established using 9 C than using previous methods.

SmC reprogramming inhibits Wnt signaling activation and TGFb in a manner similar to SmiPSC, SmNPC, SmNSs, and SmN reprogramming but is based on a wider variety of epigenetic modulators compared with those in the other cell types. According to Fu et al., SmC reprogramming is derived from the SmiPSC method; therefore, the signaling is most similar to that of SmiPSCs. Epigenetic modulation, cardiac development, and reprogramming are closely related^62^, suggesting that SmCs are induced through various epigenetic mechanisms similar to those that contribute to the production of SmiPSCs.

## Liver cells

Many researchers are studying the possibility of using hepatic stem/progenitor cells for clinical applications^63–65^. Although the liver is a regenerating organ in vivo, no conclusive evidence of a liver progenitor has been reported. The theory of a liver progenitor assumes that regeneration occurs in the parenchyma and bile ducts^66,67^ or that mature hepatocytes are reprogrammed under chronic liver injury conditions^68,69^.

On the theoretical basis that reprogramming occurs in mature hepatocytes, Katsuda et al. conducted a reprogramming study to convert mature rat and mouse hepatocytes into small-molecule-mediated bipotent liver progenitor cells (SmLPs) in vitro^70^. They studied small molecules used in previous studies of iPSC generation^24,71,72^ and found that PD0325901, Y-27632, A83-01, and CHIR99021 promoted mammary progenitor cells in culture. Therefore, to confirm the reprogramming of rat hepatocytes in vitro, they used these four small molecules in a small-hepatocyte culture medium, as reported in a previous paper^73^. In the presence of several combinations of the four small molecules, the cells proliferated, with the combination of Y-27632, A83-01, and CHIR99021 (YAC) showing the highest proliferative capacity. After 2 weeks, the number of mature rat hepatocytes treated with YAC increased ~30-fold, and the expression of proliferation markers and liver progenitor markers was upregulated. To identify the bipotential differentiation ability, an important feature of a liver progenitor, Katsuda et al. conducted hepatic differentiation experiments using a previously reported method^74^. After hepatic differentiation, the cells showed a typical mature hepatocyte form, with canaliculi structures and increased expression of mature hepatocyte markers. The team also observed functions such as albumin secretion and Cyp1a activity. They conducted a separate differentiation study for the biliary epithelial cells, which are another potential liver progenitor. These differentiated cells expressed biliary epithelial cell markers and exhibited functional characteristics and a tubular form. These cells were stably reprogrammed in six donors and cultured without losing hepatic differentiation capacity after passage 20. When the reprogrammed cells were transplanted into uPA transgenic mice crossed with severe combined immunodeficiency (SCID) mice (cDNA-uPA/SCID mice)^75^, 75–90% of the cells were repopulated without forming tumors. Although the reprogramming methods using YAC were efficient, they failed in humans.

**Fig. 2:**
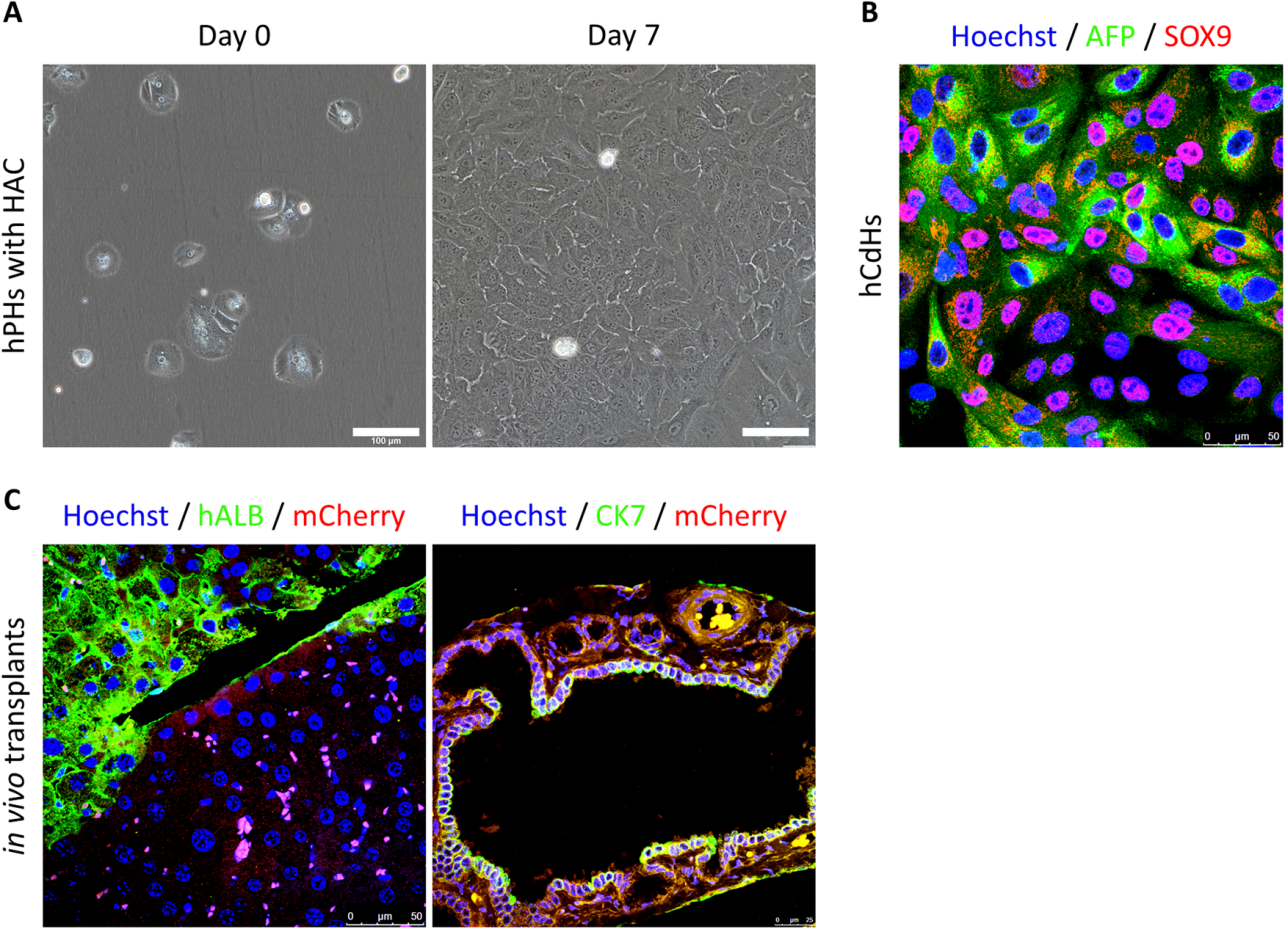
hCdH generation and in vivo transplantation of hCdHs into liver disease model mice. **a** Freshly isolated human primary hepatocytes were cultured for 7 days in HAC-containing reprogramming medium. The morphology of human primary hepatocytes (hPHs) changes rapidly in the reprogramming medium, and the capacity to proliferate is acquired. Scale bars, 100 μm. **b** After generation, human chemically derived hepatic progenitors (hCdHs) effectively express the progenitor markers alpha fetoprotein (AFP) and SOX9. Scale bars, 50 μm. **c** A liver paraffin Section 3 weeks after mCherry-tagged hCdHs were transplanted into Alb-TRECK/SCID mice. Liver sections were stained with human albumin (ALB, green, left), human cytokeratin 7 (CK7, green, right), and mCherry (red, both), and the nuclei were counterstained with Hoechst. Scale bars, 50 μm.

Drawing on the results of these studies, our group reprogrammed small molecules to transform human adult hepatocytes into SmLPs^76^. Using the cocktail and reprogramming method of Katsuda et al.^70^, we further investigated the factors that increased conversion efficiency. We found that a combination of hepatocyte growth factor (HGF), which is important for hepatic progenitor regeneration and maintenance^64,77^, A83-01, and CHIR99021 (HAC), induced reprogramming of the human hepatocytes (Fig. 2a). These chemically derived human hepatic progenitors (hCdHs) showed rapid proliferative capacity and expressed hepatic progenitor markers 10–15 days after HAC treatment (Fig. 2b). The hCdHs showed a significant increase in stem-cell–related gene set expression and were stably reprogrammed in 6 donors with maintained chromosomal integrity. Identification of the downstream signaling messengers of the HGF receptor (the MET signaling pathway) confirmed the role of HGF in generating hCdHs. As a result, we observed that, among downstream targets of MET, ERK phosphorylation was increased in the presence of HGF during the reprogramming stage. According to the results from additional experiments, we confirmed that the hCdHs were generated through an ERK1/2 mechanism. To measure the bipotent differentiation capacity of these hCdHs, differentiation experiments were carried out following the protocol found in a previous paper^74^. After hepatic differentiation, the expression of mature hepatocyte–specific genes increased significantly, and we observed that these differentiated cells had the functional characteristics of mature hepatocytes in albumin secretions and during urea synthesis and CYP1A2 activity, and upon glycogen staining. When bile epithelial cell differentiation was induced via a 3D culture system to confirm the bipotent differentiation potential of the hCdHs^78^, the hCdHs formed a tubular structure, and the expression of a biliary marker was increased. These hCdHs also maintained proliferation capacity, expression of progenitor markers, and hepatic differentiation capacity for more than 10 passages. To further characterize the hepatic differentiation of these hCdHs, we compared their global gene expression with that of other hepatocyte-like cells. The results from a clustering analysis revealed that, among hepatocyte-like cells, the hepatically differentiated hCdHs showed the highest correlation with mature hepatocytes, such as human iPSC-derived hepatocytes, ESC-derived hepatocytes, direct-converted hepatocytes, and liver progenitor-like cells. Next, we transplanted the hCdHs into Alb-TRECK/SCID mice and NOD Cg-Prkdc^scid^Il2rg^tm1Wjl^/SzJ mice (Fig. 2c). The results show that the hCdHs repopulated as much as 20% of the diseased livers, and the levels of serum human albumin and alpha-1 antitrypsin were increased after transplantation. The hCdHs differentiated into hepatocytes and biliary epithelial cells in vivo. Taken together, our results show that our HAC reprogramming system for human hepatocytes has bipotent differentiation ability in vitro and in vivo.

A comparable study by Fu et al. revealed that the reprogramming of human hepatocytes was completed within a month^79^. This group reported reprogramming SmLPs via SIRT1 signaling. They removed CD24^+^ or EpCAM^+^ cells from human hepatocytes using FACS sorting and cultured the sorted cells with transition and expansion medium (TEM) containing HGF, EGF, Y-27632, CHIR99021, A83-01, sphingosine-1-phosphate (an activator of GPR, S1P), and lysophosphatidic acid (an activator of EDG, LPA). Under TEM culture conditions, human hepatocytes were rapidly reprogrammed, and the expression of progenitor markers with normal diploid karyotypes was increased. These SmLPs could be cultured for as many as 10 passages in seven of the nine patients and survived through passage 20 with proliferation capacity in three patients. The proliferation ability was significantly reduced in the hepatocytes cultured with nicotinamide, which is known to inhibit the sirtuin protein family, and the expression of SIRT1 was increased under TEM conditions. Thus, supported with the results from additional inhibitor experiments, Fu et al. found that SIRT1 is an important factor in human hepatocytes. After hepatic differentiation, the SmLPs differentiated into mature hepatocytes with a repopulation efficiency of 7–16% when they were transplanted into Fah^−/−^Rah2^−/−^ (F/R) mice. Next, the team produced hepatitis B virus (HBV)-infected 3D spheroid models of the SmLPs in a 3D culture. Their HBV spheroids effectively expressed viral factors such as retinoid-X receptor (RXR) A, HNF4A, and the viral receptor NTCP, and they extensively characterized HBV. Additionally, this HBV model was confirmed to be reproducible in HBV patient-derived SmLPs. Ultimately, anti-HBV studies using a Cas9 system and this HBV spheroid model were possible. Reprogrammed cells can therefore be suitable cell sources for disease modeling.

Similar approaches to reprogramming human hepatocytes into SmLPSs with proliferation capacity have also been reported by Zhang et al.^80^, who modified the culture medium on the assumption that the Wnt3a, Rspo1, noggin, and FSK included in human liver isolation medium (HLIM), which also contained FGF10, EGF, HGF, human gastrin I, A83-01, Y-27632, were not optimized for the culture of human hepatocytes. They found that the proliferation of human hepatocytes was significantly reduced after the Wnt3a-conditioned medium was removed from the HLIM. Conversely, when Rspo1, Noggin, and FSK were removed from the HLIM, the proliferation of the human hepatocytes increased significantly. This result confirmed that Wnt3a is critical for the proliferation of human hepatocytes and that Rspo1, Noggin, and FSK should be removed from HLIM to enable the proliferation of human hepatocytes. Approximately 77% of the human hepatocytes cultured with modified HLIM had KI67 + cells within 3 day and maintained the expression of progenitor markers for more than a month. These proliferating cells were able to undergo hepatocyte maturation under 3D culture conditions and showed a significant increase in the expression of hepatic markers such as ALB and AAT. This reprogramming was possible in all 7 donors, with younger donors showing higher proliferation abilities. The human hepatocytes cultured in modified HLIM and transplanted into FRG mice showed a high survival rate and repopulated approximately 64% of the surviving mice. In contrast to that of our findings^76^, the histology report by Zhang et al. showed that all these cells differentiated into hepatocytes only in vivo. The results of their study suggest a way to produce proliferating cells that can differentiate into mature hepatocytes through a medium containing the Wnt3a signaling pathway components.

Parallel finding of Zhang et al., we found that HGF, which regulates cell growth, cell motility, and morphogenesis^81^, plays an essential role in the reprogramming of human hepatocytes, and Wnt-β-catenin, which is closely related to liver development and health^82^, also plays a major role in the reprogramming of SmLPs.

## Other cell types

### Adipocytes

Brown adipose tissue (BAT) has recently received attention as the source for a potential treatment for obesity and metabolic diseases^83,84^. Whereas white adipose tissue accumulates energy, BAT dissipates energy in the form of heat, which is related to the energy homeostasis of the body^85^. In 2008, *PRDM16* was reported to be a major factor in the conversion of skeletal myoblasts to small-molecule-mediated BAT-like adipocytes (SmBAs)^86^. Based on that report, Nie et al. conducted a high-throughput phenotypic screening of 20,000 compounds in skeletal myoblasts to find small molecules that induce the overexpression of PRDM16^87^. As a result, adipogenesis was observed in the cells exposed to some of these compounds, including bexarotene (a RAR agonist, Bex). Citing the report that RXR induced UCP1 in brown adipocytes^88^, the Nie team studied whether Bex/RXR causes brown adipogenesis. Their experiments confirmed that adipogenic reprogramming occurred two days after the cells were treated with Bex. The reprogrammed cells showed increased expression of Prdm16 and brown adipocyte markers such as UCP1 and suppressed white adipogenesis. Through further experiments, the group also found that *Rxrα/γ* activation is essential for the induction of BAT. To confirm the effect of Bex in vivo, they administered Bex orally to mice fed a high-fat-diet for 4 weeks, which resulted in less weight gain compared with that of a control group. In conclusion, Nie et al. reported methods that were all based on the use of small molecules to induce SmBA production in vitro and in vivo.

### β cells

The induction of the differentiation of cells from the liver in the endocrine pancreas is an attractive strategy because the liver and pancreas have the same precursors at the embryonic endoderm stage^89^. Liu et al. therefore reprogrammed rat liver epithelial stem-cell–like WB-F344 cells (WB cells) into small-molecule-mediated insulin-secreting cells (SmISCs) in three stages^90^. First, they treated WB cells with 5-aza-2′-deoxycytidine (an inhibitor of DNA methylase, 5-AZA) for 2 days, followed by trichostatin A (a regulator of chromatin remodeling, TSA) for 1 day. In the first step, cell proliferation was reduced, the cells became spindle shaped, and expression of the liver marker ceased. In the second step, they treated the cells with 1× insulin transferrin, selenite, and retinoic acid (RA) for 7 days. At that stage, the size of the cell decreased, and the gene expression of Pdx1, a pancreatic progenitor, increased. Gene expression in stage 2 was similar to the expression observed during the early pancreatic stage during development. In the final stage, the cells were treated with nicotinamide for 7 days, which produced mature SmISCs. After maturation, the cells increased their expression of islet-specific markers, and insulin genes were also activated. When these SmISCs were transplanted into streptozotocin-treated mice, the mouse glucose levels were maintained, and the serum insulin concentration more than doubled compared with that of the control group. Liu et al. therefore successfully used small molecules to reprogram hepatic stem-like cells into pancreatic progenitors expressing Pdx1 and insulin-producing cells that functioned in vitro and in vivo.

### Small molecules

**Fig. 3:**
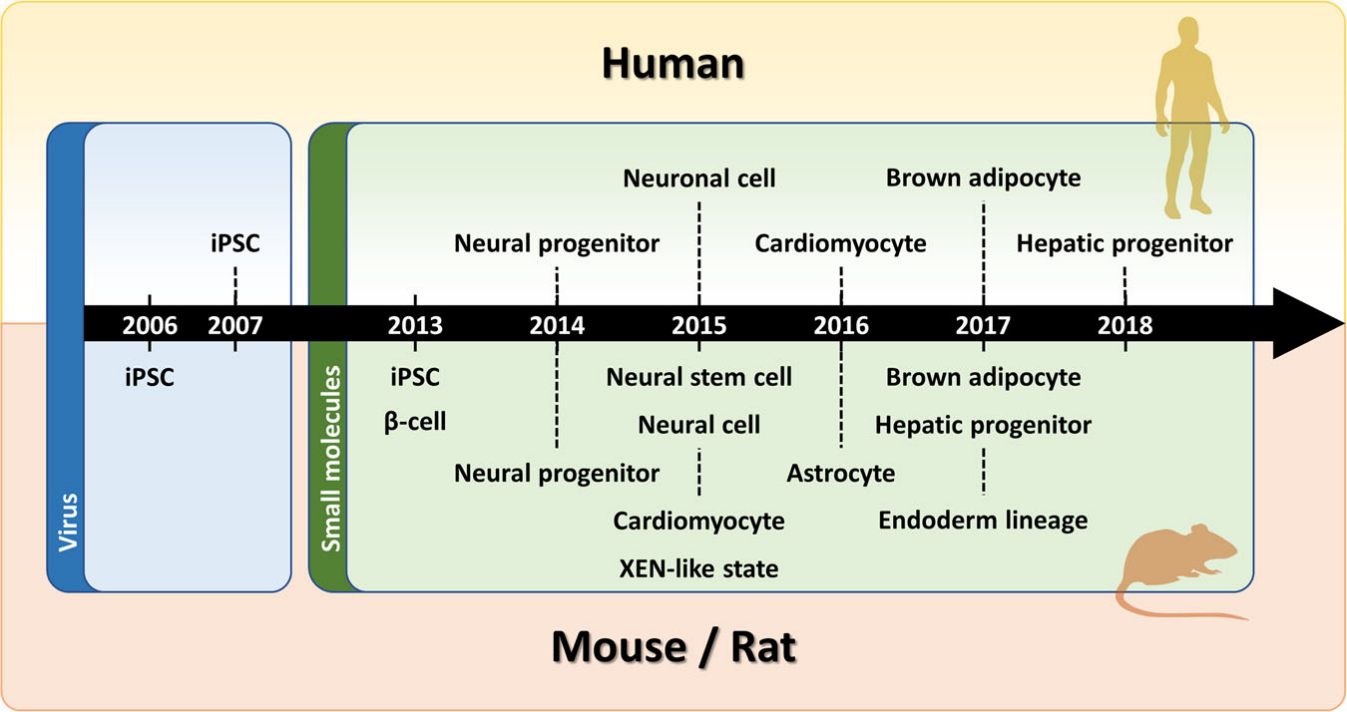
Historical timeline of small-molecule-mediated reprogramming. Reprogramming efforts using small molecules by year. In 2006, iPSCs were created using viruses (blue), and since 2013, small-molecule reprogramming (green) has been developed. The timeline is divided into mouse/rat and human phases. Immediately after the reprogramming of β cells and iPSCs was announced in 2013, the reprogramming of other cells was reported.

As discussed above, various cell types can be reprogrammed using combinations of small molecules (Fig. 3 and Table 1). The molecular and biological mechanisms of reprogramming some types of cells into other types are largely understood, but reprogramming via small molecules provides further information about these mechanisms (Table 2).

**Table 1:** Cellular reprogramming with small molecules.

Cell type	Small molecule	Source	Species	Reference
induced pluripotent stem cells	VPA, CHIR99021, 616452, Tranylcypromine	Fibroblast	Mouse	^24^
	VPA, CHIR99021, 616452, Tranylcypromine, Forskolin, DZNep, EPZ004777, Ch55, AM580	Fibroblast Neural stem cells Small intestinal epithelial cells	Mouse	^25^
Neural stem cells	SB-431542, LDN193189, CHIR99021, PD0325901, Pifithrin-α, Forskolin	Fibroblast	Mouse Human	^28^
	A83-01, LDN193189, CHIR99021, Hh-Ag 1.5, Retinoic acid, SMER28, RG108, Parnate, bFGF	Fibroblast	Mouse	^32^
Neuronal cells	CHIR99021, Dorsomorphin, Forskolin, Go6983, RepSox, SP600125, VPA, Y-27632	Fibroblast	Human	^36^
	CHIR99021, DAPT, LDN193189, Purmorphamine, SAG, SB-431542, TTNPB, Tzv, VPA	Astrocyte	Human	^37^
Cardiomyocyte	CHIR99021, Forskolin, Insulin, LIF, Parnate, PD0325901, RepSox, TTNBP, Vitamin C, VPA	Fibroblast	Mouse	^54^
	A83-01, AS8351, BIX01294, CHIR99021, JNJ-10198409, OAC2, SC1, SU16F, Y-27632	Fibroblast	Human	^59^
Hepatic progenitor	Y-27632, A83-01, CHIR99021, EGF	Hepatocyte	Mouse	^70^
	HGF, EGF, A83-01, CHIR99021	Hepatocyte	Human	^76^
	HGF, EGF, Y-27632, CHIR99021, A83-01, S1P, LPA	Hepatocyte	Human	^79^
	FGF10, EGF, HGF, human gastrin I, A83-01, Y-27632, Wnt3a	Hepatocyte	Human	^80^
Adipocyte	Bexarotene, Dexamethasone, Indomethacin, Insulin, Iso-buylmethylxanthine, T3	Skeletal myoblast	Mouse	^87^
β-cell	5-AZA, TSA, Retionic acid, Nicotinamide	Liver epithelial stem-like cells	Rat	^90^

**Table 2:** Small molecules.

Chemical component	Mechanism	Reprogrammed cells	References
616452, RepSox	Inhibitor of TGF-βRI	iPSCs	^24,25^
		Neuronal cells	^36^
		Cardiomyocytes	^54^
5-Aza-2′-deoxycytidine	DNA methyltransferase activity inhibitor	β-cells	^90^
A83-01	TGF-β RI kinase inhibitor IV	Neural stem cells	^32^
		Cardiomyocytes	^59^
		Hepatic progenitors	^70,76,79,80^
AM580	RARα agonist	iPSCs	^25^
AS8351	histone demethylase (HDM) inhibitor	Cardiomyocytes	^59^
Bexarotene	RAR agonist	Adipocytes	^87^
bFGF	Basic fibroblast growth factor	Neural stem cells	^32^
BIX01294	Histone methyltransferase inhibitor	Cardiomyocytes	^59^
Ch55	RAR agonist	iPSCs	^25^
CHIR99021	GSK-3 inhibitor	iPSCs	^24,25^
		Neural stem cells	^28,32^
		Neuronal cells	^36,37^
		Cardiomyocytes	^54,59^
		Hepatic progenitors	^70,76,79^
DAPT	γ-secretase inhibitor	Neuronal cells	^37^
Dorsomorphin	BMP inhibitor	Neuronal cells	^36^
DZNep	Histone Methyltransferase EZH2 Inhibitor	iPSCs	^25^
EGF	Epidermal growth factor	Hepatic progenitors	^70,76,79,80^
EPZ004807	DOT1L inhibitor	iPSCs	^25^
FGF10	Fibroblast growth factor 10	Hepatic progenitors	^80^
Forskolin	Adenylyl cyclase activator	iPSCs	^25^
		Neural stem cells	^28^
		Neuronal cells	^36^
		Cardiomyocytes	^54^
Go 6983	PKC inhibitor	Neuronal cells	^36^
HGF	Hepatocyte growth factor	Hepatic progenitors	^76,79,80^
Hh-Ag 1.5	Hh Signaling Pathway Agonist	Neural stem cells	^32^
human gastrin I	CCK2 receptor agonist	Hepatic progenitors	^80^
Insulin	Endogenous peptide agonist	Cardiomyocytes	^54^
JNJ-10198409	PDGFR Tyrosine Kinase Inhibitor IV	Cardiomyocytes	^59^
LDN193189	ALK2 and ALK3 inhibitor	Neural stem cells	^28,32^
		Neuronal cells	^37^
LIF	Leukemia Inhibitory Factor	Cardiomyocytes	^54^
LPA	A ligand activator for EDG-2, EDG-4, and EDG-7	Hepatic progenitors	^79^
Nicotinamide	PARP-1 inhibitor	β-cells	^90^
OAC2	Oct4 activator	Cardiomyocytes	^59^
Parnate, Tranylcypromine	Monoamine oxidase inhibitor, LSD1 inhibitor	iPSCs	^24,25^
		Neural stem cells	^32^
		Cardiomyocytes	^54^
PD0325901	Potent inhibitor of MEK1/2	Neural stem cells	^28^
		Cardiomyocytes	^54^
Pifithrin-α	p53 inhibitor	Neural stem cells	^28^
Purmorphamine	Smo receptor agonist	Neuronal cells	^37^
Retinoic acid	Endogenous retinoic acid receptor agonist	Neural stem cells	^32^
		β-cells	^90^
RG108	Non-nucleoside DNA methyltransferase inhibitor	Neural stem cells	^32^
Sphingosine-1-Phosphate	A ligand for EDG-1 and EDG-3 and activator of GPR3, GPR6, and GPR12	Hepatic progenitors	^79^
SAG	Hedgehog signaling activator	Neuronal cells	^37^
SB-431542	Inhibitor of TGF-βRI, ALK4 and ALK7	Neural stem cells	^28^
		Neuronal cells	^37^
SC1	Dual inhibition of extracellular signal-regulated kinase 1 and Ras GTPase	Cardiomyocytes	^59^
SMER28	Positive regulator of autophagy	Neural stem cells	^32^
SP600125	JNK inhibitor	Neuronal cells	^36^
SU16F	PDGFRβ inhibitor	Cardiomyocytes	^59^
Trichostatin A	Histone deacetylase inhibitor	β-cells	^90^
TTNPB	RAR agonist	Neuronal cells	^37^
		Cardiomyocytes	^54^
Thiazovivin	ROCK inhibitor	Neuronal cells	^37^
Valproic acid	Histone deacetylase inhibitor	iPSCs	^24,25^
		Neuronal cells	^36,37^
		Cardiomyocytes	^54^
Wnt3a	Wnt family	Hepatic progenitors	^80^
Y-27632	ROCK inhibitor	Neuronal cells	^36^
		Cardiomyocytes	^59^
		Hepatic progenitors	^70,79,80^

**Fig. 4:**
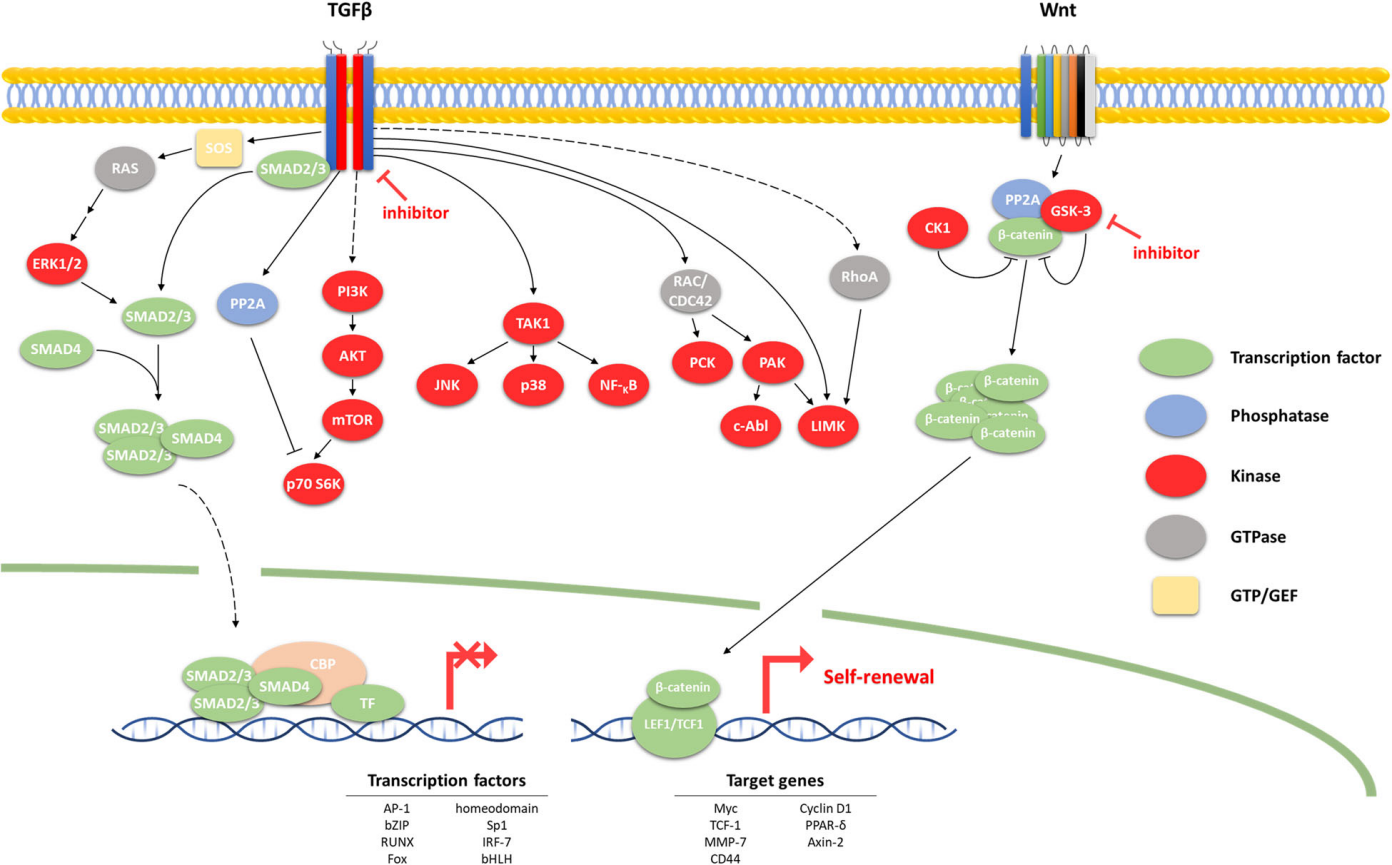
TGF-β and Wnt signaling pathways. This figure summarizes the TGF*-β* and Wnt signaling pathways, which are mechanistically leveraged to reprogram all cell types. All TGF*-β*-related signaling follow a mechanism that is suppressed by small molecules that inhibit the TGF*-β* receptor. In contrast, Wnt signaling is inhibited by *β*-catenin phosphorylation by GSK-3, but small molecules can be used to inhibit GSK-3, thereby allowing *β*-catenin to enter into the nucleus to initiate transcription. Expression of TGF-β target genes is therefore suppressed in all cell types during reprogramming, and the target genes of Wnt signaling are transcribed.

As shown in the reports presented above, CHIR99021 is used in almost all small-molecule cell reprogramming. CHIR99021 activates the Wnt signaling pathway by inhibiting GSK3^91^ (Fig. 4). During Wnt signaling, GSK-3 binds to β-catenin and degrades it via phosphorylation, inhibiting transcription. CHIR99021 selectively inhibits GSK-3, allowing β-catenin to enter the nucleus and proceed with transcription. Because the target gene of this transcription consists of genes related to the cell cycle, CHIR99021 induces self-renewal of stem cells. This process suggests that the Wnt pathway plays an important role in the induction during the primitive stage.

TGF-β inhibitors, such as 616452, A83-01, Repsox, and SB-431542, are used to reprogram almost all cells. The TGF-β inhibitors used for small-molecule-mediated reprogramming follow mechanisms that inhibit TGF-β receptors. TGF-β phosphorylates SMAD2/3, which then binds to SMAD4 and enters the nucleus to regulate transcription. TGF-β signaling plays an important role in regulating cell growth, differentiation and development, and TGF-β inhibitors are expected to confer stem-cell properties by inhibiting these regulatory pathways. Additionally, inhibition of the TGF-β pathway has been reported to replace *Sox2* and induce *Nanog* during reprogramming^30^, and several stem-cell factors are known to play important roles in regeneration^34,92^.

RAR agonists, including AM580, Bex, Ch55, RA, and TTNPB, have also been used in the reprogramming of various cells. RAR signaling plays a critical role in molecular reprogramming and leads to pluripotency through synergistic interactions^93^. It is also known to induce differentiation of stem cells, leading them to various cell fates^94,95^. Small molecules that regulate the methylation/acetylation of DNA or histones, such as 5-AZA, AS8351, BIX01294, DZNep, RG108, TSA, and VPA, are also important to reprogramming cocktails. The induction of epigenetic changes opens chromatin, activates various pathways related to reprogramming, increases the population of Oct4-positive cells, and improves reprogramming efficiency^29,96–98^. It has also been demonstrated that frequently used ROCK inhibitors reduce senescence^99^.

**Fig. 5:**
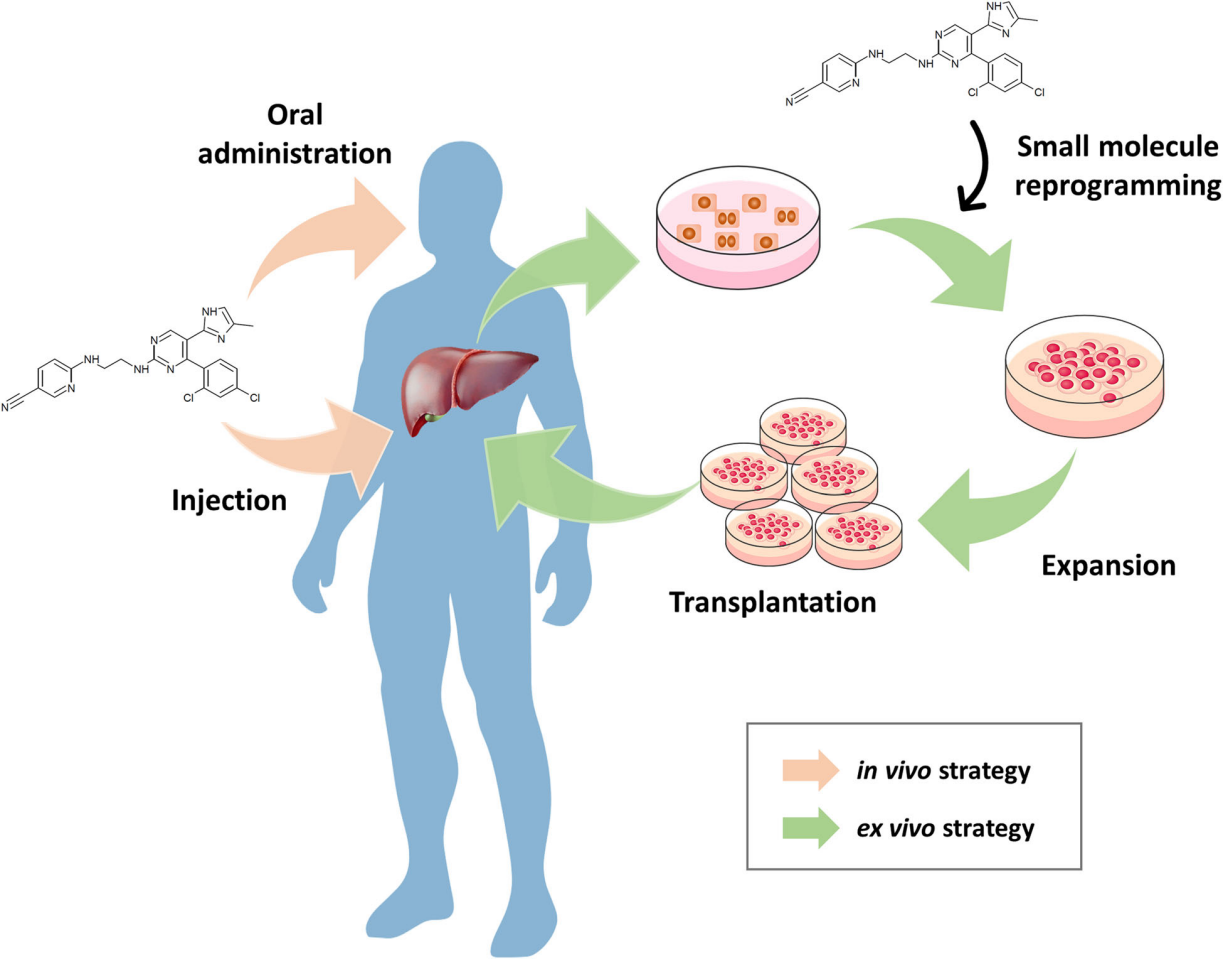
Future challenges for small-molecule-mediated clinical trials. The future direction of personalized medicine is through small molecules. An ex vivo strategy can generate the desired cells through in vitro reprogramming using small molecules, and these cells are then expanded and transplanted into patients (green arrow). The in vivo strategy is to apply a small-molecule combination directly to patients via oral administration or injection (pink arrow).

Reprogramming of functional cells using these small molecules is more likely to be applied to future regenerative medicine than other stem-cell sources (Fig. 5). The risk of tumor formation can be reduced by avoiding the problem of genetic integration posed by viruses and using existing transcription factors. Furthermore, small molecules are relatively easy to study in reprogramming in vivo and are amenable to oral administration, field injection, and intravenous injection. The next steps toward clinical application will involve confirming the possibility of inducing tumorigenicity and determining the proper concentration, combination of small molecules, and treatment times in vivo.

## Conclusion

Cellular reprogramming provides the possibility of clinical application of stem cells for drug screening, disease modeling, artificial organ development, and cell therapy. Recently, to address the issue of ectopic gene expression and virus incorporation into chromosomes, reprogramming using small molecules was introduced. However, as with other stem cells, the functional maturation of reprogrammed cells and the efficiency of their reprogramming need to be increased, and a more complete understanding of the reprogramming mechanisms is required. However, the development of small-molecule-mediated reprogramming is eventually expected to be used for cell therapy, drug screening, personalized medicine, disease modeling, and even understanding regenerative medicine.
